# Cognitive-behavioural therapy has no effect on disease activity but improves quality of life in subgroups of patients with inflammatory bowel disease: a pilot randomised controlled trial

**DOI:** 10.1186/s12876-015-0278-2

**Published:** 2015-05-02

**Authors:** Antonina Mikocka-Walus, Peter Bampton, David Hetzel, Patrick Hughes, Adrian Esterman, Jane M Andrews

**Affiliations:** 1 School of Nursing and Midwifery and Sansom Institute for Health Research, University of South Australia, Adelaide, Australia; 2 Department of Health Sciences, University of York, Area 4, ARRC Building, Heslington, YO10 5DD UK; 3 School of Psychology, University of Adelaide, Adelaide, Australia; 4 School of Medicine, Flinders University, Adelaide, Australia; 5 Department of Gastroenterology and Hepatology, Flinders Medical Centre, Bedford Park, Australia; 6 Department of Gastroenterology and Hepatology, Royal Adelaide Hospital, Adelaide, Australia; 7 Nerve-Gut Research Laboratory, Discipline of Medicine, University of Adelaide, Adelaide, Australia; 8 School of Medicine, University of Adelaide, Adelaide, Australia

**Keywords:** Cognitive-behavioural therapy, Flare, Mental health, Psychological, Quality of life, Remission

## Abstract

**Background:**

Studies have demonstrated usefulness of cognitive-behavioural therapy (CBT) in managing distress in inflammatory bowel disease (IBD); however, few have focused on IBD course. The present trial aimed to investigate whether adding CBT to standard treatment prolongs remission in IBD in comparison to standard therapy alone.

**Methods:**

A 2-arm parallel pragmatic randomised controlled trial (+CBT – standard care plus either face-to-face (F2F) or online CBT over 10 weeks versus standard care alone (SC)) was conducted with adult patients in remission. IBD remission at 12 months since baseline was the primary outcome measure while the secondary outcome measures were mental health status and quality of life (QoL). Linear mixed-effect models were used to compare groups on outcome variables while controlling for baseline.

**Results:**

Participants were 174 patients with IBD (90 +CBT, 84 SC). There was no difference in remission rates between groups, with similar numbers flaring at 12 months. Groups did not differ in anxiety, depression or coping at 6 or 12 months (p >0.05). When only participants classified as ‘in need’ (young, high baseline IBD activity, recently diagnosed; poor mental health) were examined in the post-hoc analysis (n = 74, 34 CBT and 40 controls), CBT significantly improved mental QoL (p = .034, d = .56) at 6 months. Online CBT group had a higher score on Precontemplation than the F2F group, which is consistent with less developed coping with IBD in the cCBT group (p = .045).

**Conclusions:**

Future studies should direct psychological interventions to patients ‘in need’ and attempt to recruit larger samples to compensate for significant attrition when using online CBT.

**Trial registration:**

The protocol was registered on 21/10/2009 with the Australian New Zealand Clinical Trials Registry (ID: ACTRN12609000913279).

## Background

Inflammatory bowel disease (IBD) is a chronic inflammatory condition of the gastrointestinal (GI) tract, with a rising incidence worldwide. IBD’s aetiology is thought to involve a deregulated immune response to intestinal microbiome, triggered by environmental factors, in those with a genetic susceptibility. In addition, a psycho-neuro-immunological view of IBD has developed in recent years due to intriguing observations that psychological status can directly influence inflammatory lesions in the gut [1]. Although IBD has not, to date, been confirmed to be causally linked to psychological factors, psychological stress has been reported to be one of the strongest predictors of symptomatic disease course [2,3] and also linked to increased inflammation [4]. A review by Graff et al. has demonstrated that depression negatively influences IBD course [5]. However, it needs to be acknowledged that in the majority of the available studies, reported symptoms rather than actual inflammation (measured e.g. using fecal calprotectin or histology scores) are the outcome measure and thus it remains unclear how much of this relationship is explained by symptom perception versus inflammatory activity. Further to this, although the research on stress, depression and inflammation in animals has provided more than convincing evidence of their inter-relationships in IBD, replicating these observations in humans is challenging for ethical reasons. Hence, the question of this relationship needs to be addressed indirectly by testing whether delivering a therapy known to enhance psychological wellbeing (i.e. psychotherapy) may protect against IBD-related inflammation and prevent flares.

Given the observed relationship between stress, depression and IBD disease course and also the encouraging data on the treatment of functional gut disorders with psychotherapy [6], interest in the possible efficacy of psychological interventions in IBD has been growing. In recent years, several reviews have been published on the efficacy of psychotherapy for QoL, emotional status and remission/flare status in IBD [7-10]. A Cochrane meta-analysis of randomized controlled trials found no evidence for the efficacy of psychotherapy in improving QoL, emotional status or proportion of patients not in remission in the short term or at 12 months in unselected adults with IBD, although it suggested that psychotherapy may result in benefits for subgroups of patients [8]. However, here, ideologically dissimilar psychological interventions were grouped together possibly clouding the conclusions. One of the recent reviews [9] has addressed this weakness of the previous review and concluded that cognitive-behavioural therapy (CBT) - a psychotherapy where people are taught to identify and modify unhelpful thinking styles and maladaptive behaviours - is associated with improvement in distress, although it has resulted in benefits with respect to IBD symptoms only in some reports.

Thus, the present trial aimed to investigate whether CBT in addition to standard medical therapy prolongs remission in IBD in comparison with standard therapy alone. As IBD is a life-long condition [11] and there is the potential for a bidirectional interaction between psychological status and disease relapse [12], and the additional factor that life stresses and psychological status are not constant over time, we chose to examine the effect of CBT in a general cohort of IBD patients in remission rather than only those with current depression and/or anxiety. Given the primary outcome measure and the main focus of the trial was on disease activity over time rather than treating anxiety or depression, this was considered the most optimal approach.

It was hypothesised that the benefits of adding CBT would be two-fold:

i) increase in the proportion of IBD patients remaining free of a flare in comparison to standard therapy and
ii) increase in health-related quality of life (QoL).

## Methods

### Design

The study was a 2-arm parallel randomised controlled trial (RCT) (standard care plus CBT (+CBT) versus standard care alone (SC)). The trial design was pragmatic as it offered those in the experimental group a choice of completing the intervention face-to-face (F2F) or online (cCBT). The psychological intervention was not compared with time-matched psychological placebo as CBT has previously been found to have a significant effect over and above the placebo in many studies [13]. In this real world study, SC (involving additional visits to the clinic to give blood and complete the questionnaires at baseline, 6 and 12 months) was the preferred comparator as this is the current best practice. The study was registered in the Australian New Zealand Trial Registry (ACTRN12609000913279). The reporting of this study followed the CONSORT statement format in relation to pragmatic trials [14]. Partial concealment was used at invitation/enrolment as SC patients were told that we were examining how psychological health influenced IBD behaviour and were unaware of the +CBT arm of the study. *Blinding* the experimental group was not possible as is often the case in psychotherapy trials.

### Participants

Participants were recruited from two Gastroenterology Clinics in Australia, together serving approximately 2,000 IBD patients.

#### Inclusion criteria

Patients had to meet ALL of the following criteria: 1). a clinically established diagnosis of IBD (according to usual clinical practice by combination of clinical, radiologic, endoscopic and histologic grounds in a tertiary care IBD centre); 2). current clinical remission or mild symptoms only for at least 3 months as evidenced by disease activity index, notes review, blood results and report from their treating gastroenterologist, if necessary (complete loss of GI symptoms in IBD is uncommon even during endoscopic remission); 3). sufficient English to understand, answer questionnaires and participate in therapy; 4). 18 years old or older; 5). competence to consent; 6). willingness to complete CBT sessions.

#### Exclusion criteria

Patients were excluded if they met ANY of the following: 1). serious mental illness (e.g. psychosis, schizophrenia) or alcohol/substance dependence as diagnosed by the Clinical Psychologist; 2). currently undergoing psychotherapy; 3). significant cognitive impairment. Antidepressants were not an exclusion as antidepressants are commonly used in IBD not necessarily to treat anxiety or depression but rather, in a similar fashion they are used in functional gut disorders, to manage pain and abdominal discomfort and thus may be considered usual care [15].

### Procedure

IBD databases were screened by the Clinics’ IBD nurses. Potentially eligible IBD patients were then contacted by a letter. Further approaches were made to patients who did not opt-out or answer the letter within 4 weeks, up to three times. Patients from outside of the hospitals who were known to the clinics’ doctors were contacted by their own treating doctors by letters. Eligible patients were then randomised to either the +CBT or SC group. A simple randomisation method was used using a table of computer generated random numbers (in blocks of four) in the proportion of 2:1 (experimental vs. control). This proportion was used as we predicted problems in recruiting to the experimental arm (due to a larger participant burden) and assumed that we needed to approach twice as many experimental participants to eventually obtain the same number of participants in both groups. A randomisation schedule was created by the researcher with no direct patient contact using computer software (AE). Participants were enrolled by Research Nurses (not this study’s investigators) who also assigned participants to interventions.

The +CBT arm had two subgroups which for the purpose of the main analysis were treated as one: a F2F CBT group that met at the hospital; and a cCBT group where intervention was delivered online. Those who could not commit to the time/travel involved in F2F CBT were offered therapy online which was designed to exactly mirror the F2F intervention. F2F group met weekly at the hospital for two-hour sessions delivered by a psychologist while cCBT group received sessions of similar length online (self-directed). Sessions are presented in detail elsewhere (http://www.tameyourgut.com/); their content was identical for both experimental sub-groups. Irrespective of CBT allocation, F2F and cCBT groups both reported for blood tests and assessments at the same times. See Table 1 for assessment points. Reminders about the next session were sent via email or text message weekly for the duration of the intervention for both CBT subgroups.

**Table 1:** **Measurements at time points**

	Baseline	6 months	12 months
**SC**	Demographics Blood test, IBD activity, QoL measure, Mental health measures	Blood test, IBD activity, QoL measure, Mental health measures	Blood test, IBD activity, QoL measure, Mental health measures
**CBT**	Demographics Blood test, IBD activity, QoL measure, Mental health measures	Blood test, IBD activity, QoL measure, Mental health measures	Blood test, IBD activity, QoL measure, Mental health measures

### Intervention

CBT was a 10-week group program designed specifically for this patient population by senior clinical psychologists working at the hospital Clinical Psychology Unit (not study investigators). This length is consistent with the maximum annual number of psychotherapy sessions with a psychologist currently available to Australians as part of the Medicare Better Access initiative [16]. Based on previous studies [17,18] we assumed that CBT would perform consistently irrespective of delivery mode. The CBT program (2 hours each week) consisted of: 1) Education about IBD and CBT; 2) Stress and relaxation; 3) Automatic thoughts and cognitive distortions; 4) Cognitive restructuring; 5) Exposure and overcoming avoidance; 6) Coping strategies; 7) Assertiveness training; 8) Relationships and communication; 9) Attention and distraction; and 10) Relapse prevention for mental health problems. Attendance at each weekly session was noted by the psychologist and registered by the CBT website in the case of online groups. *Compliance* with the program was monitored by the psychologist on a regular basis and strategies to minimise *attrition* included regular reminders (telephone, email). Non-compliant patients were contacted by the psychologist individually and inquired about their views on how this may be improved. Withdrawing patients were asked for permission to retain data to date. *Treatment fidelity* was maintained by using the same protocol for both groups within the CBT arm. *Online CBT* was provided using the university IT infrastructure support.

### Sample size

The power calculation was prepared for the primary outcome measure being in remission at follow-up for the two main groups (experimental and control). Specifically, with 80 patients per arm, the study would have 80% power at the 0.05 level to detect a 20% difference in the proportion of patients remaining in remission between the +CBT group (0.80) and SC (0.60) arms. This was based on average data for remission duration and rates of IBD from several studies [19-24] and what was thought to be a clinically relevant significant difference of 20% as judged by the investigators and based on data from studies on CBT in irritable bowel syndrome, as no reliable estimates from previous studies on IBD and CBT were available [6]. We intended to recruit approx. 192 participants to allow for drop-outs of 20%.

### Measurements

*Disease activity* was scored with the Crohn’s Disease Activity Index (CDAI) [25] for those with CD or the Simple Clinical Colitis Activity Index (SCCAI) [26] for those with UC as appropriate. Disease activity was also monitored using blood tests: CRP, HB, platelet, WCC. The Short Form 36 Health Status Questionnaire (SF-36) was used to measure *Health-Related Quality of Life* (HRQOL) [27,28]. It is a comprehensive questionnaire which yields an 8-scale health profile, and summary measures of HRQOL (Physical component score, PCS and Mental component score, MCS). *Mental health* was assessed using the Hospital Anxiety and Depression Scale (HADS), the State-Trait Anxiety Inventory (STAI), the Brief COPE, the Revised Social Readjustment Rating Scale (RSRRS) and the IBD Stages of Change Coping Questionnaire (IBDSCCQ). The HADS is a self-assessment mood scale developed for medical outpatients [29] containing 14 questions graded on a 4-point Likert scale, with subscales of anxiety and depression, with a sum score ranging from 0 to 21 for each. The STAI [30] is used to differentiate between temporary condition of “state anxiety” and the more general and long-standing quality of “trait anxiety”. The scale has 40 items, scored on a 1–4 scale. The Brief COPE [31] is a short 28-item questionnaire, scored on a 4-point Likert scale and derived from a widely used COPE scale. The questionnaire identifies a number of adaptive and maladaptive coping styles. The RSRRS [32] measures 43 stressful events, with scores interpreted as follows: Low stress <149; Mild stress = 150–200; Moderate stress = 200–299; Major stress >300. The IBDSCCQ is a short 10-item questionnaire designed by these investigators in order to monitor changes in the process of thinking about coping with IBD. It is as yet not validated. The questionnaire was derived from the work by Carr [33] and previous work of the second investigator [34] in the Trans-Theoretical Model of behavioural change (TTM). The TTM presents individuals as progressing through five stages involving decisions about change [35]. The stages comprise: Precontemplation, Contemplation, Preparation, Action and Maintenance. High scores on Precontemplation imply undeveloped coping with IBD and every subsequent stage corresponds with improved coping, thus high scores on Maintenance are consistent with excellent coping with IBD.

### Outcome measures

IBD remission at 12 months (established using the CDAI score of <150 or the SCCAI score of >3 and confirmed by the treating physician) was the primary outcome measure in this trial; secondary outcome measures were quality of life as measured on the SF-36 and mental health status (anxiety and depression as measured on the HADS and the STAI, coping as measured on the Brief COPE and the IBDSCCQ, and stress as measured on the RSRRS) at 6 and 12 months.

### Statistical analysis

The study applied the intention-to-treat analyses for the main group comparisons at 6 months and per protocol analyses in the CBT dose–response group comparisons. Participants ‘in need’ were defined as meeting at least one of the following criteria: being young and thus recently transitioned from paediatric care (aged 18–20 years); having high baseline IBD activity despite being considered in IBD remission by the clinician (CDAI >180; baseline SCCAI >5); being recently diagnosed (within last 2 years); having poor coping (a score of 20–25 on either adaptive or maladaptive coping); and high anxiety or depression (HADS score for either anxiety or depression subscale >/= 15). Descriptive statistics including means, SD, counts and proportions were used to describe the study population in the two treatment arms at baseline, 6 and 12 months. Univariate group comparisons were conducted with Mann–Whitney test and the chi-square test. Within group changes over time were calculated with the Friedman test. The multivariate analyses were conducted using the linear mixed-effects models which allowed compensating for the missing data. Two models were constructed: model 1 included time and group variables and a time-group interaction variable and adjusted for the outcome variable at baseline; model 2 also adjusted for sex and age. The p value of less than .05 was considered statistically significant. Data were analysed using PASW (SPSS, Inc., Chicago, IL).

### Ethical considerations

The study was approved by the Royal Adelaide Hospital and the University of South Australia Research Ethics Committees in August 2009. The trial was conducted following the principles of the Declaration of Helsinki (updated in 2008). Participants gave written informed consent and the confidentiality was ensured.

## Results

**Figure 1:**
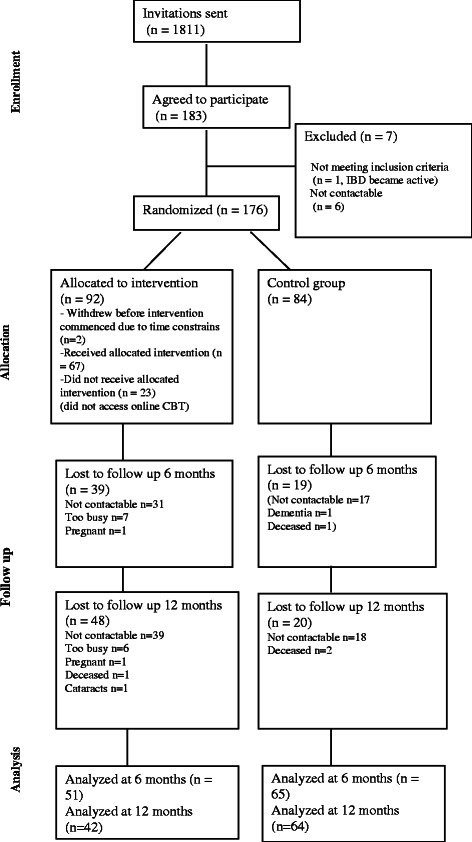
CONSORT diagram showing the flow of participants through the study.

Patient recruitment is presented in Figure 1. Overall, 174 patients participated in the study: 90 in the +CBT group and 84 in SC.

### Baseline demographics, clinical history, medication use, disease activity, psychological scores, CBT use and ‘in need’ status

Patient demographics and clinical history are presented in Table 2. CBT participants were more commonly younger and female. Medication use is presented in Table 3. Blood results are presented in Table 4.

**Table 2:** **Demographic characteristics and clinical history by group**

		+CBT	SC
		n = 90	n = 84
		n (%)	
**Gender**	**Male**	40 (44.4)	54 (64.3)
**Speaks language other than English at home**	**Yes**	5 (5.6)	6 (7.1)
**Marital status**	**Married/de facto**	59 (65.6)	50 (59.5)
**Employment status**	**Full-time work**	38 (42.7)	33 (39.3)
	**Part-time work**	16 (18)	15 (17.9)
**Education**	**Year 12 completed**	28 (31.1)	21 (25)
	**Bachelor’s degree**	23 (25.6)	17 (20.2)
**IBD subtype**	**Crohn’s disease**	58 (64.4)	49 (58.3)
**Hospital admissions for IBD**	**Yes**	52 (60.5)	50 (61)
**Operations for IBD**	**Yes**	43 (48.9)	31 (37.8)
**Suffers from other chronic illness**	**Yes**	48 (53.9)	49 (61.3)
		**Mean (SD)**	
**Age**	**In years**	46.5 (15.7)	51.9 (16.9)
**Years with IBD symptoms**		16.1 (12.1)	14.3 (11.7)
**Years since diagnosis**		11.8 (10.4)	11.7 (11.8)
**Number of IBD specialists**		2.6 (1.6)	2.6 (1.9)
**How long with current specialist**	**In years**	5.7 (6.2)	5.5 (6.1)
**How long since your last flare**	**In years**	2.6 (3.9)	2.8 (6.6)
**How many hospital admissions for IBD**	**Last 5 years**	4.7 (3.8)	4.7 (3.6)

**Table 3:** **Medication use**

	+CBT			SC		
	n (%)					
	Baseline	6 months	12 months	Baseline	6 months	12 months
	n = 90	n = 51	n = 42	n = 84	n = 65	n = 64
**Azathioprine**	44 (49)	21 (41)	16 (38)	22 (26)	16 (25)	16 (25)
**Mesalazine**	25 (28)	11 (22)	10 (24)	32 (38)	20 (31)	22 (34)
**Sulphasalazine**	13 (14)	6 (12)	7 (16)	14 (17)	12 (18)	10 (15)
**Corticosteroids**	10 (11)	6 (12)	3 (7)	8 (9)	5 (8)	6 (9)
**Adalimumab**	10 (11)	7 (14)	2 (5)	6 (7)	4 (6)	4 (6)
**Infliximab**	10 (11)	8 (16)	5 (12)	5 (6)	5 (8)	6 (9)
**Vedolizumab**	3 (3)	1 (2)	1 (2)	1 (1)	0	0
**Methotrexate**	4 (4)	4 (8)	2 (5)	4 (5)	2 (3)	2 (3)
**Mercaptopurine/thiopurine**	2 (2)	2 (4)	5 (12)	4 (5)	0	5 (8)
**Vitamins**	28 (31)	12 (23)	6 (14)	23 (27)	12 (18)	11 (17)
**Fish oil**	17 (19)	12 (23)	9 (21)	11 (13)	9 (14)	6 (9)
**Probiotics**	11 (12)	5 (10)	5 (12)	7 (8)	6 (9)	6 (9)
**Paracetamol**	24 (27)	14 (27)	10 (24)	18 (21)	10 (15)	11 (17)
**Codeine**	10 (11)	3 (6)	4 (9)	4 (5)	4 (6)	3 (5)
**Ibuprofen**	6 (7)	2 (4)	2 (5)	3 (4)	1 (1)	1 (1)
**Dextropropoxyphene**	4 (4)	1 (2)	0	1 (1)	1 (1)	0
**Antispasmodics**	2 (2)	1 (2)	1 (2)	1 (1)	2 (3)	4 (6)
**Morphine**	1 (1)	0	0	2 (2)	0	1 (1)
**Oxycodone hydrochloride**	1 (1)	3 (6)	2 (2)	2 (2)	3 (5)	0
**Tramadol**	1 (1)	0	0	1 (1)	0	0
**Naproxen**	0	1 (2)	0	0	0	0
**Medication for depression**	24 (28)	14 (29)	10 (24)	20 (27)	12 (18)	12 (19)

**Table 4:** **Blood results by group**

	+CBT			SC		
	Mean (SD)					
	Baseline	6 months	12 months	Baseline	6 months	12 months
	n = 69	n = 57	n = 54	n = 70	n = 62	n = 61
**CRP**	3.6 (5.4)	5.2 (12.7)	4.9 (10.8)	6.2 (8.3)	10.8 (27.6)	6.5 (10.8)
**HB**	136.6 (21.8)	136.4 (14.8)	136.6 (13.9)	141.9 (15.4)	142.3 (17.2)	141.3 (17.1)
**Platelet**	260.9 (72.8)	260.8 (64.7)	267.1 (67.1)	266.4 (63.2)	272.1 (74.8)	263.5 (76.3)
**WCC**	5.9 (1.8)	8.3 (14.8)	6.1 (1.9)	6.6 (2.1)	6.9 (2.5)	6.9 (2.5)

#### Disease activity

Although all patients enrolled were considered to be in IBD remission or to have only mild symptoms by their treating team, patients’ subjective report seemed to correspond poorly with formal activity scores (Table 5). Only 13 (7 CBT and 6 SC) participants considered their IBD control poor, yet at baseline, 18 UC participants had SCCAI scores >3 and 26 CD participants had CDAI >150.

**Table 5:** **Disease activity at baseline and 6 months as measured on the patient subjective measure of disease activity and disease activity indices**

		+CBT			SC		
		n (%)					
		Baseline	6 months	12 months	Baseline	6 months	12 months
		n = 90	n = 51	n = 42	n = 84	n = 65	n = 64
**IBD control**	**Very well**	27 (30)	18 (35)	14 (33)	18 (21.4)	17 (26.1)	27 (42)
	**Reasonably**	54 (60)	26 (50)	18 (43)	56 (66.7)	35 (53.8)	30 (47)
	**Not well**	7 (8)	3 (6)	5 (12)	6 (7.1)	7 (10.8)	3 (5)
**CDAI**	**Active >150**	15 (17)	8 (16)	5 (12)	11 (13.1)	8 (12.3)	8 (12)
**SCCAI#**	**Active >3**	8 (9)	8 (16)	6 (14)	10 (11.9)	14 (21.5)	9 (14)
		**Mean (SD)**					
**CDAI**		110.8 (72.5)	91.3 (90.3)	84.1 (95.3)	87.4 (104.8)	83.2 (92.2)	88.4 (105.9)
**SCCAI**		3.4 (1.4)	3.5 (1.8)	2.9 (1.6)	3.2 (1.3)	3.7 (1.6)	3.5 (2.5)

#Contrary to the commonly used cut off point of >2, in this study SCCAI was considered normal between 0 and 3 as only few participants (all considered by doctors to be in remission) had a score below 3 with subscore of 0 for rectal bleeding.

#### Other outcome variables

Both groups reported high levels of stress (>300) (Table 6). However, they also tended to have low scores on the IBDSCCQ subscale of Precontemplation and high scores in Maintenance, which corresponds with already advanced coping with IBD.

**Table 6:** **Group differences on mental health variables**

		+CBT			SC		
		Baseline	6 months	12 months	Baseline	6 months	12 months
		n = 90	n = 51	n = 42	n = 84	n = 65	n = 64
		Mean (SD)					
**Physical QoL**		46.7 (9.3)	46.7 (10.3)	48.3 (10.3)	47 (10.3)	47.2 (9.8)	48.3 (10.1)
**Mental QoL**		44.8 (11.4)	48.3 (9.2)	45.5 (11.1)^1^	48.1 (11.5)	48.1 (11.9)	48.3 (11.5)
**HADS Anxiety**		7.1 (3.9)	5.9 (3.4)	6.5 (4.2)	6.2 (4.3)	5.9 (4.6)	6.1 (4.6)
**HADS Depression**		4.3 (3.4)	3.5 (2.9)	4.1 (3.3)^2^	4.4 (4.1)	4.4 (4.1)	4.5 (4.8)
**State Anxiety**		37.5 (13.1)	34.5 (10.9)	35.9 (13.1)	35.9 (13.7)	35.9 (12.8)	35.3 (13.4)
**Trait Anxiety**		39.3 (11.9)	36.1 (11.1)	39.5 (12.5)^3^	37.4 (11.7)	37.5 (11.8)	36.9 (13.1)
**Adaptive coping**	*Range: 20–80 (higher is better)*	42.7 (12.8)	42.1 (13.6)	40.5 (12.9)	39.5 (11.3)	37.1 (11.2)	37.5 (11.7)
**Maladaptive coping**	*Range: 8–32 (lower is better)*	10.9 (3.6)	9.8 (2.6)	10.6 (3.7)^4^	10.7 (3.7)	10.5 (3.3)	10.5 (3.6)
**Stress**	*>300 high stress 150–299 moderate stress <150 low stress*	638.3 (665.9)	474.2 (653.7)	301.3 (347.5)^5^	453.6 (490.5)	350.7 (403.4)	338.5 (364.2)^6^
**TTM Stage**	**Pre-contemplation**	4.5 (1.6)	4.3 (1.9)	4.2 (1.9)^7^	4.4 (1.6)	4.2 (1.9)	4.7 (1.9)
*Range:2–10 (higher scores mean greater agreement)*							
	**Contemplation**	6.7 (2.1)	6.6 (1.9)	6.6 (1.6)	6.3 (2.1)	6.2 (2.1)	6.4 (1.9)
	**Preparation**	6.3 (2.3)	6.5 (2.4)	6.4 (2.3)	5.7 (2.3)	6.1 (2.2)	6.1 (2.1)
	**Action**	5.8 (2.2)	6.3 (2.3)	6.1 (2.3)	5.5 (2.1)	5.8 (2.3)	5.9 (2.3)
	**Maintenance**	7.1 (1.9)	7.7 (1.7)	7.3 (1.8)	7.2 (1.3)	7.1 (1.9)	7.3 (1.9)

^1^A significant improvement in mental QoL over 12 months in the +CBT group (chi^2^ (2) = 8.63, p = .013).

^2^A significant improvement in HADS Depression over 12 months in the +CBT group (chi^2^ (2) = 8.02, p = .018).

^3^A significant improvement in Trait Anxiety over 12 months in the +CBT group (chi^2^ (2) = 6.32, p = .042).

^4^A significant improvement in maladaptive coping over 12 months in the +CBT group (chi^2^ (2) = 12.12, p = .002).

^5^A significant drop (improvement) in Precontemplation over 12 months in the +CBT group (chi^2^ (2) = 6.29, p = .043).

^6^A significant improvement in stress over 12 months in the +CBT group (chi^2^ (2) = 11.04, p = .004).

^7^A significant improvement in stress over 12 months in the SC group (chi^2^ (2) = 8.71, p = .013).

#### Mode of CBT delivery and its usage

Of 90 CBT participants, 67 (74.4%) used CBT (either face-to-face or online, at least one CBT activity completed) and 23 (25.6%) did not use it at all. Of CBT users, 44 (48.8%) used a reasonable amount of CBT program (>4 sessions face-to-face or at least 20 online logins to activities) while 23 (25.5%) used a </=4 sessions face-to-face or <20 online activities. Those who used more CBT, had lower mental QoL at baseline (p = .032), higher depression scores (p = .019) but were less stressed at baseline than those who used less (p = .012). Overall, 68 participants opted to use cCBT and 22 were allocated to F2F CBT. cCBT users did not differ in age or sex from F2F users (p >.05).

#### Participants ‘in need’

In the whole sample, there were 74 (42.5%) participants ‘in need’: 34 in the +CBT and 40 in the SC group. Those ‘in need’ in the +CBT and SC groups did not differ significantly at baseline on any of the variables of interest.

### Primary outcome measure

At 12 months, 30 (73.2%) +CBT and 43 (71.7%) SC participants remained in remission as measured on disease activity indices (p = .868). At the multivariate level (adjusting for baseline), CBT did not significantly change disease activity at 12 months (CD, p = .669; UC, p = .549). Controlling for sex and age did not alter the results.

### Secondary outcome measures

While at the univariate level +CBT group significantly improved in mental QoL, depression, trait anxiety, maladaptive coping, Precontemplation and stress, and the SC group only in stress, between baseline and 12 months (Table 6), at the multi-variate level, CBT did not significantly affect these measures (all p >.05). Further adjusting for sex and age did not change the results significantly.

### Additional analyses

#### Changes in blood results

At the multi-variate level, CBT did not significantly affect the CRP, the WCC, the HB or the platelet levels at 6 or 12 months (all p >.05). Further adjusting for sex and age did not change the results significantly.

#### CBT dose–response

No relationship between dose of CBT and all but one outcome measure was found (p >0.05). When CBT users (at least one CBT activity completed) were compared to non-users (in the CBT group but never accessing CBT) in a multivariate model, the groups were found different on the IBDSCCQ subscale of Contemplation at 6 months (p = .020, d = .94). At 12 months the groups were found different on Contemplation when adjusted for sex and age (p = .030, d = .66). CBT users had a higher score of contemplation than non-users, reflecting improvement in coping with IBD resulting from CBT use.

#### F2F versus cCBT

While at the univariate level F2F group improved significantly in two dimensions of coping (maladaptive coping and Precontemplation) and cCBT in stress (Table 7), at the multivariate level, no relationship between mode of CBT delivery and all but one outcome measure was found (p >0.05). When controlled for sex and age, at 12 months, the cCBT group had a higher score on the IBDSCCQ subscale of Precontemplation than the F2F (4.7 (SD = 1.8) vs. 3.3 (SD = 1.9), p = .045, d = .75), which is consistent with less developed coping with IBD in the cCBT group.

**Table 7:** **Outcome variables by mode of CBT delivery**

		F2F				cCBT	
		Baseline	6 months	12 months	Baseline	6 months	12 months
		n = 22	n = 16	n = 15	n = 68	n = 35	n = 27
		Mean (SD)					
**Physical QoL**		43.2 (11.1)	41.1 (13.4)	43.5 (12.1)	47.7 (8.4)	49.2 (7.7)	50.9 (8.4)
**Mental QoL**		42.3 (10.8)	48.1 (7.7)	43.8 (10.7)	45.5 (11.5)	48.4 (9.9)	46.4 (11.4)
**HADS Anxiety**		7.8 (3.1)	6.2 (2.0)	6.7 (2.7)	6.7 (4.2)	5.8 (3.8)	6.4 (4.8)
**HADS Depression**		5.1 (2.7)	4 (2.5)	4.7 (2.9)	4.1 (3.6)	3.3. (3.1)	3.7 (3.5)
**State Anxiety**		41.1 (12.9)	36.1 (10.8)	38.3 (10.1)	36.4 (13.1)	33.8 (11.1)	34.6 (14.5)
**Trait Anxiety**		43.3 (11.2)	36.3 (13.7)	42.2 (10.2)	38.1 (11.9)	36 (9.7)	37.9 (13.5)
**Adaptive coping**	*Range: 20–80 (higher is better)*	44.1 (10.3)	45 (13.6)	46.1 (12.5)	42.2 (13.5)	40.6 (13.6)	37.6 (12.4)
**Maladaptive coping**	*Range: 8–32 (lower is better)*	11.8 (2.7)	10.3 (1.9)	11.4 (3.4)^1^	10.6 (3.8)	9.5 (2.9)	10.2 (3.8)
**Stress**	*>300 high stress 150–299 moderate stress <150 low stress*	660.4 (606.1)	755.8 (934.2)	406.1 (471.6)	630.7 (689.6)	345.5 (434.7)	240.9 (241.5)^2^
**TTM Stage**	**Pre-contemplation**	4.6 (1.3)	3.5 (1.4)	3.3 (1.9)^3^	4.5 (1.7)	4.6 (2.1)	4.7 (1.8)
*Range:2–10 (higher scores mean greater agreement)*							
	**Contemplation**	7.1 (1.9)	7 (1.7)	6.8 (1.9)	6.5 (2.1)	6.5 (1.9)	6.5 (1.4)
	**Preparation**	7.2 (1.9)	7.4 (2.1)	7.3 (2.1)	6 (2.3)	6.2 (2.5)	5.9 (2.4)
	**Action**	6.8 (2.1)	7.3 (2.1)	7.1 (2.1)	5.4 (2.1)	5.8 (2.3)	5.5 (2.3)
	**Maintenance**	7.6 (1.6)	8 (1.2)	7.5 (1.7)	6.9 (2.1)	7.6 (1.8)	7.2 (1.9)
**CDAI**		140.3 (87.1)	75.3 (83.3)	79.5 (111.6)	101.2 (65.5)	97.3 (93.7)	86.9 (88.2)
**SCCAI**		3.7 (1.1)	4.3 (1.4)	4.0 (1.1)	3.2 (1.5)	3 (1.9)	2.3 (1.5)

^1^A significant improvement in maladaptive coping between baseline and 12 months in the F2F group (chi^2^ (2) = 7.20, p = .027).

^2^A significant improvement in stress between baseline and 12 months in the cCBT group (chi^2^ (2) = 8.79, p = .012).

^3^A significant drop (improvement) in Precontemplation between baseline and 12 months in the F2F group (chi^2^ (2) = 12.3, p = .002).

#### Participants ‘in need’

Those participants ‘in need’ who received CBT had improved mental QoL at 6 months as compared to SC (p = .034, d = .56). At 12 months the difference disappeared, however, the group ‘in need’ was very small at this time point (n = 43, +CBT n = 11 and SC n = 32) and the comparison clearly underpowered (d = .26).

### Attrition

At 6 months, 39 (43%) CBT group participants and 19 (23%) SC were lost to follow up (p <.001) indicating a higher drop-out rate in the CBT group. The overall attrition at 6 months was 33%. Attrition at 6 months was similar in the cCBT versus F2F group (33 (48%) vs. 6 (27%), p = .080). At 12 months, 48 (53%) CBT group participants and 20 (24%) SC were lost to follow up (p <.001). The overall attrition at 12 months was 39%. Attrition at 12 months was higher in the cCBT versus F2F group (41 (60%) vs. 7 (32%), p = .020).

## Discussion

This trial is the largest to date to explore the potential usefulness of CBT in managing disease activity and mental health in IBD. A pragmatic approach to its delivery was chosen, offering participants a choice between face-to-face and online CBT, designed to mirror each other.

The most important finding of the study is that adding CBT to standard care was not found protective of IBD remission over a period of 12 months. Further to this observation, participants who received CBT were not found to differ in their scores on anxiety, depression and coping measures at 6 months when controlling for baseline, a finding in contrast to previous small studies [36-40] and unusual given the high efficacy of CBT in treating anxiety and depression [41]. It can perhaps be explained by low anxiety and depression scores at baseline, and thus since the mental health of these participants was generally good at the outset, there was little that could be gained from the intervention. A subgroup analysis of participants ‘in need’ (with high scores on mental health subscales), showed that CBT was effective in improving this group’s QoL at 6 months, thus confirming that CBT works in those who need it. At 12 months, due to attrition, the comparison was underpowered and thus future studies should establish long-term effect of CBT on QoL in the subgroups of IBD classified as ‘in need’. A study using psychodynamic therapy for a similar unselected sample also showed no relationship between psychotherapy and disease activity in IBD, although unlike in our study, theirs showed trends towards improved disease activity and fewer surgical interventions [42]. On the other hand Keefer et al. [43] showed that gut-directed hypnotherapy prolongs clinical remission in IBD. Though Keefer et al.’s study [43] had a small sample size, their promising results may indicate that hypnotherapy rather than therapies such as CBT or psychodynamic may play a role in maintenance of IBD remission.

Regarding the participants ‘in need’, this group was identified based on the recommendations from previous papers [8,44]. Indeed, looking at this subgroup separately, we showed that CBT does improve mental QoL at least at short-term. Curiously, no improvements in anxiety or depression were observed in the group ‘in need’, however, given its size (34 CBT and 40 controls), it is possible that if the sample had been larger these improvements would have appeared and possibly a longer observation period could also verify this finding. Future studies should focus on targeting psychotherapeutic interventions at the group ‘in need’ rather than the general IBD population and should confirm these results on a larger sample to allow for significant attrition.

Interestingly, this trial did not show F2F to be significantly different to cCBT in efficacy, with the exception of one dimension of coping (showing F2F group to fare better at 12 months), although, admittedly the trial was not powered for this subgroup analysis. Participants nearly always preferred the online CBT delivery as it fitted with their life style and commitments and thus it is reassuring that the efficacy of the modalities was similar. This mode of delivery also allowed for recruiting participants who usually do not take part in trials as they live in remote areas, increasing the generalisibility of our data. However, the attrition in the online CBT group was significantly larger than in the F2F group and the ways to address high attrition should be examined in future studies. Alternatively larger samples could be utilised.

Further, and interestingly, we observed no marked relationship between the amount of CBT used and the outcomes. Only when we compared users to non-users (in the CBT group but not using the program) we showed some difference in the IBDSCCQ subscale of Contemplation at 6 and 12 months, demonstrating that those who used CBT improved their coping with IBD. However, we failed at demonstrating that a particular dose of CBT is effective as all the comparisons between users and controls were not statistically significant.

Finally, while this trial is the largest to date, it unavoidably has limitations. Its design does not comply with the classic RCT requirements in which participants are aware they may be offered an intervention or the placebo. Given impossibility of blinding the intervention, we decided to withdraw the information regarding the intervention from the controls. Thus, the controls had been informed they participated in an observational study on mental health in IBD. Further, having extensive clinical experience with this population who is in the productive age, often in full-time employment but also frequently caring for young families, we gave participants the option of choosing the mode of CBT delivery. If the study was to be conducted using the classical RCT design, we should have randomly allocated participants to each mode of delivery. In addition, while we conducted the analysis of CBT usage, there is no certainty that our statistics on the online CBT usage are 100% reflective of the actual use. A significant number of patients, particularly, the older ones, preferred to print the materials and work on them offline and thus the short time spent on a given activity may not be a reflection of it not being read or acted on. Perhaps because of it we did not show a significant relationship between CBT usage and the outcomes. Disease activity throughout the trial was measured using disease activity indices and monitoring blood. Future studies could use calprotectin and/or endoscopy to monitor disease activity, although the latter would likely lead to low participation and drop-out due to participant burden. Using calprotectin was not possible in this trial as calprotectin had not been available at the participating hospitals when we commenced the study and the study only received a limited funding. Further, the sample size calculation was prepared for the main group comparisons only and the study was not powered for the subgroup analyses. However, no good estimates for what to base the subanalysis predictions on had been available and the future trials can use the present study data to provide adequate power calculations. Finally, although the attrition in this trial was approximately 30% at 6 months and nearly 40% at 12 months, significantly more CBT users than control group participants withdrew from the study, possibly reflecting the study burden. However, high attrition is a known phenomenon in the self-directed psychotherapy studies for those in the intervention group [18,45], and thus our study does not differ in respect to this from other research in the field.

## Conclusions

CBT does not seem to improve disease activity at 12 months since baseline in the unselected patients with IBD although it offers promise to patients ‘in need’. Future studies should direct psychological interventions to patients ‘in need’ and attempt to recruit larger samples to compensate for significant attrition when using online CBT. Longer follow-up is needed to clarify any potential longitudinal effect of CBT in IBD management.

## Abbreviations

+CBT: Standard care plus CBT
CBT: Cognitive-behavioural therapy
CCBT: Online CBT
CD: Crohn’s disease
CDAI: Crohn’s disease activity index
CRP: C-reactive protein
F2F: Face-to-face
GI: Gastrointestinal
HADS: Hospital Anxiety and Depression Scale
HB: Haemoglobin
HRQOL: Health-related quality of life
IBD: Inflammatory bowel disease
IBDSCCQ: The IBD Stages of Change Coping Questionnaire
MCS: Mental component of the SF36
PCS: Physical component of the SF36
QoL: Quality of life
RSRRS: The Revised Social Readjustment Rating Scale
SC: Standard care
SCCAI: Simple Clinical Colitis Activity Index
SD: Standard deviation
SF-36: The Short Form 36 Health Status Questionnaire
STAI: State-Trait Anxiety Inventory
TTM: The Trans-Theoretical Model of behavioural change
UC: Ulcerative colitis
WCC: White cell count
